# Effects of White Noise on Academic Skills in Children with ADHD and Specific Learning Disorders: New Perspectives for Personalised Rehabilitation and Educational Intervention

**DOI:** 10.3390/pediatric18030081

**Published:** 2026-06-11

**Authors:** Elena Cavalieri, Emilia Cascio, Giada Iannone, Loredana Angelini, Giovanni Battista Dell’Isola, Claudio Maura, Raimondo Stefano Maria Torcisi, Elisa Macchione, Simona Lucibello, Alberto Verrotti, Federico Vigevano

**Affiliations:** 1 Department of Developmental Disabilities, Istituto di Ricovero e Cura a Carattere Scientifico (IRCCS) San Raffaele, 00163 Rome, Italy; elena.cavalieri@sanraffaele.it (E.C.); loredana.angelini@sanraffaele.it (L.A.); elisa.macchione01@gmail.com (E.M.); simonalucibello1988@gmail.com (S.L.); federico.vigevano@sanraffaele.it (F.V.); 2 Speech and Language Therapy Degree Programme, Faculty of Medicine and Surgery, Università Cattolica del Sacro Cuore, 00168 Rome, Italy; ggiadaiannone@gmail.com; 3 Department of Civil Engineering and Computer Engineering, Faculty of Medical Engineering, University Tor Vergata, 00133 Rome, Italy; claudiomaura@me.com; 4 Department of Neuromotor Rehabilitation and Rehabilitation Robotics, Istituto di Ricovero e Cura a Carattere Scientifico (IRCCS) San Raffaele Roma, 00163 Rome, Italy; raimondo.torcisi@sanraffaele.it; 5 Pediatric Unit, Department of Maternal and Child Health, University of Perugia, 06123 Perugia, Italy; alberto.verrottidipianella@unipg.it

**Keywords:** rehabilitation, ADHD, specific learning disorder, white noise, reading, writing

## Abstract

Background/Objectives. This study examined whether exposure to white noise improves reading and writing performance in children with Specific Learning Disorder (SLD), with and without comorbid Attention-Deficit/Hyperactivity Disorder (ADHD). Methods. Thirty children aged 8–13 years (mean age = 9.4) with SLD, 12 of whom also had ADHD, were recruited from the Centro di Riabilitazione San Raffaele Pisana (Rome). Each child completed two standardized reading and writing assessments, four weeks apart, under two auditory conditions (with vs. without white noise) in randomized order. The primary outcomes were reading speed and accuracy, while the secondary outcome was writing accuracy. Results. Among the 26 completers, white noise significantly improved nonword reading speed and accuracy, showed a trend toward improved passage-reading accuracy, and reduced accuracy in nonword writing. Benefits were different in children with SLD + ADHD compared to those with SLD only. Conclusions. These findings indicate task-specific effects of white noise and suggest potential applications for targeted educational interventions.

## 1. Background

Attention-Deficit/Hyperactivity Disorder (ADHD) is a neurodevelopmental disorder characterized by a persistent pattern of inattention and/or hyperactivity–impulsivity that interferes with functioning or development [1].

The worldwide prevalence of ADHD in children and adolescents under 18 years ranges from 2% to 7.6%, with a mean of about 5% [2,3,4,5]. In Italy, prevalence is reported to be approximately 1% [6,7].

Although the neuropsychological profile of ADHD is highly variable, the disorder is commonly associated with deficits in several executive-function (EF) domains, particularly attention and working memory (WM) [8,9,10]. Because WM and attention are essential for temporarily storing information and maintaining focused mental activity, deficits in these domains can negatively affect learning [11,12,13,14]. Learning is a higher-order cognitive function that relies on distributed neural networks and engages multiple cognitive processes, including EFs [13,15].

The cognitive load, and thus the demands on EFs, increase markedly when children begin formal schooling [16]. Indeed, memory and attention are among the strongest predictors of later academic achievement [17,18].

ADHD frequently co-occurs with Specific Learning Disorders (SLD), with reported comorbidity rates ranging from 31% to 45% [19,20]. Research indicates that this high co-occurrence is not solely due to common EF deficits [21] but rather stems from shared genetic influences across neurodevelopmental disorders, particularly with respect to the inattentive symptom dimension [22,23].

According to the Diagnostic and Statistical Manual of Mental Disorders, Fifth Edition (DSM-5), SLD is characterized by persistent deficits in one or more academic domains, with performance below that expected for age and intellectual functioning, impairing school, work, or everyday activities [1]. SLDs encompass a cluster of clinical conditions, including dyslexia, dysorthography, dysgraphia, and dyscalculia, which frequently co-occur [17,24]. Global prevalence estimates range from 5% to 15% [25,26], whereas in Italy they range between 2.5% and 3% [13]. Attention and WM deficits are observed in SLD even in the absence of comorbid neurodevelopmental disorders [17,27,28].

Both reading and writing processes place substantial demands on WM and attention. In reading, WM supports the temporary storage of phonological and semantic information during decoding and comprehension, while attention regulates the allocation of cognitive resources to maintain accuracy and fluency. In writing, these functions are essential for planning, transcription, and error monitoring.

Specific training programs targeting WM and attention in preschool children have been shown to enhance concentration and the use of effective strategies for later school learning [16,29,30,31]. Among such interventions, recent studies suggest that exposure to white noise can improve attention and WM performance in preschool and school-aged children with ADHD [32,33,34,35,36,37,38,39,40,41].

White noise is a continuous, aperiodic auditory signal that contains all frequencies audible to the human ear with equal intensity, spanning approximately 20 Hz to 22 kHz [35,36].

White-noise therapy has previously been used to enhance concentration and cognitive abilities, improve sleep, and reduce hyperactivity-related problems by promoting relaxation in various populations [37].

This approach is based on the idea that noise can restore the cortical under-arousal observed in children with ADHD, thereby optimizing cognitive performance [36,40,42]. The phenomenon underlying these findings is known as stochastic resonance, a process in which a moderate level of noise adds signal to a subthreshold stimulus so that the detection threshold is reached, compensating for reduced behavioural functions [40]. Stochastic resonance has been documented in biological systems in both animals and humans and is thought to underlie a range of cognitive benefits [40].

Because attention and WM form the foundation of reading and writing processes, white noise might positively influence these skills [43]. However, while some studies have reported beneficial effects of white noise on attention and WM in children with ADHD, the efficacy of such treatment in children with SLD with and without ADHD lacks robust demonstration in the current literature [37].

Evidence on the cognitive effects of white noise is mixed, largely because stochastic resonance interacts with individual baseline arousal: children with predominantly inattentive symptoms (under—aroused) may benefit more, whereas those with hyperactive–impulsive traits (optimally or over-aroused) may show no improvement or even decrements in performance [36,44]. While previous research has primarily focused on the effects of white noise on general EF or isolated ADHD symptoms, its direct impact on specific academic skills—such as reading and writing- remains largely unexplored. Furthermore, it is currently unknown whether these potential benefits differ between different clinical profiles, specifically children with SLD versus those with comorbid SLD and ADHD. To address this gap, the unique contribution of the present study lies in its application of white noise directly during standardized reading and writing tasks using a crossover design. Therefore, the present study aims to evaluate whether exposure to white noise improves academic performance (reading and writing) in children with SLD, with or without ADHD.

## 2. Materials and Methods

The study was approved by the Ethical Committee Lazio Area 5 (prot. n. 7231 of 23 May 2024) and conducted in accordance with the Declaration of Helsinki. All parents of participating children provided written informed consent to authorize the assessments and to ensure privacy protection. The consent form explained the study procedures, guaranteed anonymity of personal data, and specified the non-harmful nature of white-noise exposure.

Nevertheless, possible risks related to white-noise exposure, including transient auditory or sensory discomfort, fatigue, reduced tolerance to continuous sound, or attentional overload during task performance, were considered. To minimize these risks, white noise was delivered at a moderate intensity, and a 2–3 min adaptation period was provided at the beginning of each session.

## 3. Design

We conducted a randomized crossover clinical trial comprising five sequential phases. Each participant completed two standardized assessment sessions, one with white noise and one without, in a randomized order, separated by a four-week interval. The four-week interval between the two assessment sessions was selected to minimize immediate carryover and short-term familiarity effects between conditions.

- Phase I (Recruitment and Screening): Potential participants were identified and assessed for eligibility at the Centro di Riabilitazione San Raffaele Pisana in Rome.
- Phase II (Diagnostic Grouping): Eligible children were assigned to one of two diagnostic groups based on their clinical profile (Group SLD + ADHD or Group SLD-only).
- Phase III (Randomized Sequencing): Within each diagnostic group, participants were randomly allocated to one of two exposure sequences to counterbalance order effects.
  (White-noise → No-noise, or No-noise → White-noise).
- Phase IV (First Assessment Session): Standardized assessment under randomized conditions, with or without white noise.
- Phase V (Second Assessment Session): Standardized assessment, conducted four weeks later, with or without white noise according to the previous randomization.

## 4. Sample, Inclusion and Exclusion Criteria

Recruitment was proposed for 30 patients undergoing rehabilitative therapy at Centro di Riabilitazione San Raffaele Pisana in Rome (Italy) between May 2024 and May 2025.

Patients were selected according to the following criteria:

### 4.1. Inclusion Criteria

- Intelligence Quotient (IQ) assessed using the Wechsler Intelligence Scales for Children–Fourth Edition, Italian version (WISC-IV; Giunti Psychometrics, Florence, Italy), ≥70.
- Age between 8 and 13 years.
- Diagnosis of SLD, with or without comorbid ADHD, according to DSM-5 criteria.

### 4.2. Exclusion Criteria

- Genetic disorders or sensory deficits.
- Pharmacological treatments that could interfere with task performance (including methylphenidate or specific ADHD treatments). Since stimulants optimize baseline cortical arousal to reduce inattention [45], adding white noise could induce over-arousal and worsen performance [38].

### 4.3. Drop out

Included patients could withdraw from the study at any time for the following reasons:

- Desire to leave the study;
- Refusal to cooperate with study investigators;
- Medical conditions that, in the investigator’s judgment, contraindicate continuation in the study.

Recruited patients were divided into two groups based on diagnosis: Group A (children with ADHD comorbid with SLD) and Group B (children with SLD only). After forming the two groups, randomization was performed to determine which participants would be assessed first under white-noise conditions and which would perform the first assessment in the absence of white noise.

Randomization was carried out using the Wichmann-Hill pseudo-random number generator implemented in SPSS Statistics, version 26.0. The allocation sequence was generated before enrolment by a researcher who was not involved in participant recruitment or outcome assessment. The randomization sequence was stored in sequentially numbered, opaque, sealed, and stapled envelopes.

**Figure 1:**
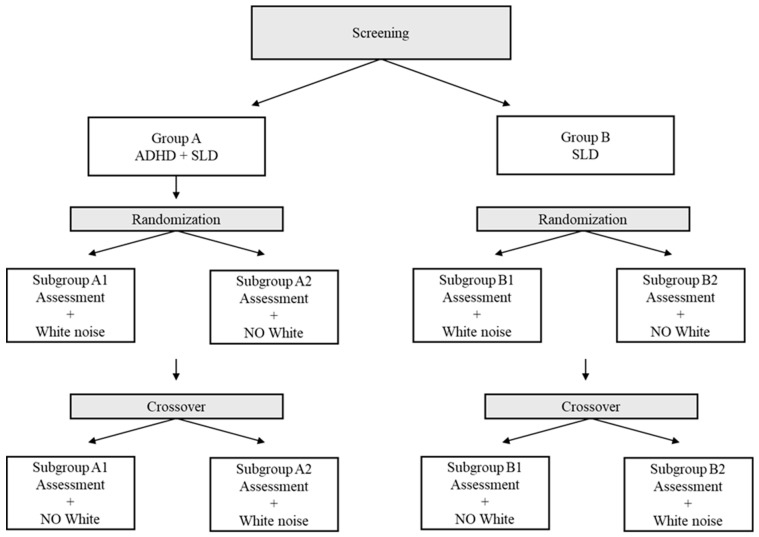
Study design flowchart.

This procedure allowed the formation of subgroups A1, A2, B1, and B2, as illustrated in the following flowchart (Figure 1).

## 5. Setting

Assessments for each child were conducted in a dedicated, identical therapy room at Centro di Riabilitazione San Raffaele Pisana for all sessions. The room contained a table with two chairs, positioned face-to-face, one for the examiner and one for the participant, to facilitate direct interaction while maintaining a comfortable interpersonal distance, and a Bluetooth speaker used to deliver white noise. The room was acoustically insulated from external noise sources, and environmental conditions (temperature, lighting and absence of external visual/auditory stimuli) were kept strictly constant across all participants and sessions.

## 6. Measures

### 6.1. White Noise

The white noise, created for this study by an audio engineer, was played through a Bose SoundLink Mini Bluetooth speaker (Bose Corporation, Framingham, MA, USA) positioned consistently at a fixed distance of 1 m from the participant, at an intensity of 60–65 dB within the designated testing environment. Before each assessment session, the sound level was checked and verified in the testing room at the participant’s seating position (at ear level) to ensure that white-noise intensity remained within the predefined range, using a calibrated digital sound level meter. At the start of each session, participants were informed of its presence to prevent any potential discomfort. This adaptation phase lasted approximately 2–3 min.

### 6.2. Passage Reading Test

The passage-reading tasks from the Prove MT-3-Clinica (Giunti Psychometrics, Florence, Italy) [46] for primary and lower-secondary school were administered to assess reading speed and accuracy. Each child was presented with a grade-appropriate passage, selected according to the norms provided in the test manual to ensure comparability with age-matched peers. The passages vary in length, lexical complexity, and syntactic structure to reflect the typical reading demands for each grade level. Administration followed the standardized procedure: the examiner instructed the child to read the passage aloud at a natural pace, without skipping or correcting words unless prompted by the protocol.

Reading speed was recorded in seconds from the first to the last word, up to a maximum of four minutes and accuracy was scored by noting the number and type of errors.

### 6.3. Word and Nonword Reading and Dictation Tests

The Batteria per la Valutazione della Dislessia e della Disortografia Evolutiva—2 (DDE-2); (Giunti Psychometrics, Florence, Italy) [47] was used to assess specific components of reading and writing performance.

Four subtests were selected: subtest 2 (word-list reading), subtest 3 (nonword-list reading), subtest 6 (word dictation), and subtest 7 (nonword dictation). In the reading subtests, participants were presented with lists of either real words or pronounceable nonwords printed in standard font and size and were instructed to read them aloud as quickly and accurately as possible. The examiner recorded both the total number of errors and the total time required to complete each list, measured in seconds with a digital stopwatch. In the writing subtests, the examiner dictated either real words or nonwords at a controlled pace, following the standardized administration protocol. Only orthographic errors were scored.

This battery provides separate normative scores for speed and accuracy in reading and for accuracy in writing.

### 6.4. Passage Dictation

Passage dictation was evaluated using the Batteria per la Valutazione Clinica della Scrittura e della Competenza Ortografica—3 (BVSCO-3); (Giunti Psychometrics, Florence, Italy) [48].

The passage-dictation task requires the transcription of a short, grade-appropriate text dictated by the examiner at a controlled pace, with pauses and repetitions following the standardized administration protocol. The difficulty of the passage is scaled according to the child’s school grade, ensuring that lexical and syntactic complexity are developmentally appropriate.

The task allows for the identification and classification of different types of spelling errors, including phonological, orthographic, and morphosyntactic errors, as defined in the scoring manual. Administration followed a standard paper-and-pencil format, and participants were permitted to write in either cursive or print, according to their usual classroom practice. All responses were scored for accuracy, with error counts and types recorded to provide a detailed profile of the child’s orthographic competence.

## 7. Outcome

For the passage-reading test and the word- and nonword-reading tasks, two outcome measures were considered: reading speed, expressed in syllables per second (syl/s), and reading accuracy, defined as the total number of errors produced. Reading speed was calculated by dividing the total number of syllables in the stimulus by the total reading time, measured in seconds.

For the passage-, word-, and nonword-dictation tasks, the outcome measure was writing accuracy, operationalized as the total number of orthographic errors. Error types were classified according to the scoring criteria of each standardized test (e.g., phoneme–grapheme correspondence errors, omissions, additions, morphological errors). Higher error counts indicated lower performance in the respective skill domain.

## 8. Statistical Analysis

Variables were summarized as mean ± SD and median [IQR]. Within-subject comparisons between the white-noise and no-noise conditions were performed for each outcome. The normality of paired differences was assessed with the Shapiro–Wilk test; when normality was not supported, we used the two-sided Wilcoxon signed-rank test, otherwise the paired Student’s *t*-test. For each outcome, we reported the mean difference (white-noise vs. no-noise conditions), its 95% confidence interval, and the effect size (Cohen’s d for paired data). To account for the crossover design, we fitted a linear mixed-effects model with a random intercept for subject and fixed effects for condition (white-noise vs. no-noise), period (second vs. first session), and sequence (order of exposure). Model assumptions were evaluated by inspecting the distribution of residuals and residual-versus-fitted plots. As an exploratory control for multiple testing, *p*-values were adjusted using the Benjamini–Hochberg false discovery rate (FDR). Statistical significance was set at two-sided *p* ≤ 0.05. Analyses were conducted with IBM SPSS Statistics, version 26.0 (IBM Corp., Armonk, NY, USA).

## 9. Results

A total of 30 children meeting inclusion and exclusion criteria were enrolled; four (13.3%) did not attend the second assessment and were excluded from paired analyses, yielding a final analytic sample of 26 participants (10 females, 38.5%; 16 males, 61.5%) with a mean age of 9.4 years (range 8–13). To assess potential attrition bias, completers (*n* = 26) and non-completers (*n* = 4) were descriptively compared on available baseline variables. No meaningful differences were observed in age, sex distribution, or diagnostic group.

With respect to schooling, 25 children (96.2%) were in primary school, and 1 (3.8%) was in lower secondary school (Table 1). Based on clinical evaluation, 12 children were classified as SLD with comorbid ADHD (Group A) and 14 as SLD-only (Group B).

Baseline comparability between crossover sequences was also examined. Participants allocated to the white-noise-first sequence (*n* = 18) and those allocated to the no-noise-first sequence (*n* = 8) did not show meaningful differences in age, sex distribution, or diagnostic subgroup.

In the full cohort (Table 2), white noise produced task-specific changes in reading and spelling. For nonword reading speed, performance was significantly faster with white noise: the mean within-subject difference was +0.140 syllables/s (95% CI 0.0437–0.2371, *p* = 0.0066, and Cohen’s d^z^ = 0.586). For nonword reading accuracy, children made significantly fewer errors under white noise: the mean difference was −2.731 errors (95% CI −4.514 to −0.948, *p* = 0.0042, and d^z^ = −0.619). Passage-reading speed and accuracy did not reach statistical significance (paired *p* = 0.051). By contrast, nonword spelling accuracy worsened significantly under white noise: children produced more errors, with a mean increase of about +2.04 errors (95% CI 0.91–3.17; paired *p* = 0.001; d = 0.73). Examination of crossover diagnostics indicated a clear period effect, with generally better reading speeds at the second session consistent with practice, whereas sequence (order) effects and evidence of carryover were not detected, supporting the adequacy of the randomized counterbalancing. After controlling for multiple testing with Benjamini–Hochberg FDR, the reduction in nonword reading errors and the increase in nonword spelling errors remained statistically significant, whereas the speed gain in nonword reading was not statistically significant after adjustment. Mixed-effects models that explicitly adjusted for period and sequence corroborated the principal findings: the condition effect was statistically significant for nonword reading errors (β ≈ −2.46, *p* = 0.009) and for nonword spelling errors (β ≈ +2.13, *p* < 0.001), while the estimate for nonword reading speed was also statistically significant (β ≈ +0.087, *p* = 0.045).

Subgroup analyses revealed distinct response profiles aligned with diagnostic status (Table 3 and Table 4). In Group A (SLD + ADHD, *n* = 12), white noise significantly reduced nonword reading errors (mean −4.25 errors; *p* = 0.004) and passage reading errors (mean −2.2 errors; *p* = 0.022), with no significant changes in spelling.

In Group B (SLD-only, *n* = 14), there was a statistically significant increase in nonword reading speed (mean +0.131 syllables/s; *p* = 0.039), accompanied by a statistically significant increase in nonword spelling errors (mean +2.07 errors; *p* < 0.001).

Direct between-group comparisons did not reach statistical significance across the outcomes; however, descriptive trends suggested descriptive differences in reading error reduction for Group A, speed gains for Group B, and spelling worsening for Group B.

## 10. Discussion

Recent studies have revealed that exposure to white noise can enhance attention and working memory in children with ADHD [32,33,34,35,36,37,38,39,40,41]. These two cognitive functions represent fundamental prerequisites for reading and writing processes, although evidence of white noise’s direct impact on these academic skills remains limited [43].

The present study contributes to this limited body of research by examining the effects of white noise on reading and writing performance in children with SLD, both with and without comorbid ADHD, using a randomized crossover design.

In the overall sample, exposure to white noise was associated with better nonword reading, with gains in speed and accuracy, and with a detriment in nonword spelling accuracy (more errors). For passage reading, accuracy did not reach statistical significance (*p* = 0.051 in inferential tests). These findings suggest that the effect of white noise might be task-specific rather than generalized. In sublexical and passage-reading tasks, which place higher demands on attention and WM, white noise appears to have a positive effect. This finding is consistent with the stochastic resonance hypothesis, which proposes that a moderate level of noise can compensate for the cortical hypoarousal typically observed in children with ADHD diagnosis, thereby improving cognitive performance [40]. However, evidence in this field remains mixed, and alternative mechanisms—such as general arousal modulation or improved attentional engagement—cannot be excluded. By contrast, in nonword writing tasks, the auditory stimulus may induce cognitive overload, interfering with phoneme–grapheme conversion and auditory discrimination, leading to an increase in errors [43]. This finding aligns with research indicating that the benefits of white noise are not uniform across cognitive domains and may depend on the complexity and sensory modality of the task [44]. Indeed, writing (particularly under dictation) requires the integration of auditory processing, orthographic retrieval, and motor execution, all of which may be more susceptible to interference from continuous background noise.

Analyses by diagnostic subgroup revealed differential sensitivity. In children with SLD + ADHD, the most consistent benefit of white noise was a reduction in nonword reading errors, accompanied by convergent improvements in other reading measures, while nonword spelling accuracy significantly worsened. This is consistent with the notion that individuals with attentional deficits may be more likely to benefit from noise-based interventions, as the added stimulation may help normalize arousal levels and optimize cognitive performance. In children with SLD only, no improvements in accuracy emerged; however, a statistically significant increase in nonword reading speed was observed. This suggests that, even in the absence of clinically significant attentional deficits, white noise may facilitate the fluency of decoding processes, possibly by enhancing temporal processing or reducing internal distractibility, without necessarily improving accuracy.

These findings have several implications. From a clinical perspective, they suggest that white noise could be considered a promising target for further investigation as a low-cost, non-invasive adjunct to traditional interventions for reading difficulties, particularly in children with SLD comorbid with ADHD. However, its use should be tailored to the specific task and individual profile, as benefits in reading may be accompanied by decrements in writing performance.

Several limitations should be considered. The study was exploratory, as no reliable a priori power calculation could be performed due to the lack of previous studies using the same population, standardized Italian academic measures, and a crossover white-noise exposure design. The relatively small, single-centre sample, gender imbalance between groups, and exclusion of children receiving ADHD medication may limit generalizability. In particular, the small sample size increases the risk of unstable estimates, Type II errors, inflated effect sizes, and limited reproducibility of findings. In addition, blinding to the auditory condition was not feasible, and although the crossover design included a four-week interval and counterbalanced exposure order, period effects were observed, suggesting possible practice or familiarity effects. Finally, the study focused on short-term standardized reading and dictation outcomes and did not include long-term follow-up or real-world educational measures.

Taken together, these findings indicate that the efficacy of white noise depends on both task demands and neurocognitive profile: appearing potentially advantageous for sublexical decoding, and most impactful in children with SLD + ADHD, but counterproductive for spelling tasks, especially in SLD-only. However, in light of the aforementioned limitations, these results must be interpreted strictly as preliminary, task-specific and hypothesis-generating. Rather than providing evidence that white noise interventions are ready to be implemented in educational or clinical settings, this pilot study serves as a foundational proof of concept. Extensive replication using multicentre, adequately powered samples, robust allocation concealment and real-world ecological measures is strictly required before any generalizable conclusions or practical recommendations can be made. From a clinical perspective, white noise may represent a promising avenue for future investigation as a low-cost, non-invasive adjunct to reading interventions, best applied in decoding-focused activities and tailored to the learner, while being avoided or used cautiously during dictation/spelling tasks.

## 11. Conclusions

In conclusion, the findings of the present study suggest that exposure to white noise does not appear to produce a generalized effect on reading and writing skills but rather shows selective effects depending on the type of task and the child’s neurocognitive profile. In the overall sample, white noise was associated with increased accuracy of nonword reading in unadjusted analyses, though speed gains did not survive multiple testing correction, while significantly reducing accuracy in nonword writing. Subgroup analyses revealed that, among children with ADHD comorbid with SLD, the benefits were evident mainly in the accuracy of nonword reading and in passage-reading accuracy, although accompanied by a statistically significant decline in nonword writing accuracy. In children with SLD only, a statistically significant improvement was observed in nonword reading speed, with no gains in accuracy and a statistically significant decline in sublexical writing accuracy.

From a clinical perspective, these findings should be interpreted with caution and do not yet support direct clinical application, although they may inform future research on strategies to support homework completion, particularly in children with attentional difficulties. It should also be noted that writing performance in the present study was assessed through dictation tasks rather than spontaneous writing; therefore, future research should further explore this aspect, as well as reading comprehension, to provide a more comprehensive understanding of white noise effects. Further studies with larger samples are needed to confirm these findings and to clarify the mechanisms underlying the differential response to white noise.

## Figures and Tables

**Table 1:** Demographic and Clinical Characteristics of the Study Sample.

	Overall Sample (*n* = 26)	ADHD + SLD (*n* = 12)	SLD (*n* = 14)
Age (M ± SD)	9.4 ± 0.99	9.6 ± 1.21	9.3 ± 0.80
Gender, N (%)			
Male, N (%)	16 (61.5%)	11 (91.7%)	5 (35.7%)
Female, N (%)	10 (38.5%)	1 (8.3%)	9 (64.3%)
Grade Level, N (%)			
Primary School (%)	25 (96.2%)	11 (91.7%)	14 (100%)
Middle School (%)	1 (3.8%)	1 (8.3%)	0 (0%)

Note: Values are presented as mean ± standard deviation (SD) or number (percentage). ADHD = Attention-Deficit/Hyperactivity Disorder; SLD = Specific Learning Disorder.

**Table 2:** Primary outcomes in the overall cohort.

	White Noise Mean ± SD and Median [IQR]	No Noise Mean ± SD and Median [IQR]	*p* Value
passage reading test speed (syl/s)	2.17 ± 1.03 1.89 [1.46–2.99]	2.03 ± 0.90 1.98 [1.33–2.74]	0.142
passage reading test errors (n°)	7.85 ± 3.68 7.50 [5.25–10.63]	9.13 ± 4.36 9 [5.63–10.50]	0.051
word reading test speed (syl/s)	2.03 ± 0.98 1.82 [1.36–2.92]	1.87 ± 0.89 1.83 [1.23–2.50]	0.051
word reading test errors (n°)	7.62 ± 6.20 6.50 [2–12]	8.69 ± 7.69 7.00 [3.25–13.75]	0.257
non-word reading test speed (syl/s)	1.35 ± 0.59 1.18 [0.91–1.98]	1.21 ± 0.50 1.09 [0.83–1.60]	**0.006**
non-word reading test errors (n°)	7.38 ± 6.18 6.50 [3–9.75]	10.12 ± 5.64 10 [6–12.75]	**0.004**
word dictation test errors (n°)	6.62 ± 6.68 4.5 [2–8]	6.73 ± 6.03 4.5 [3–8.75]	0.471
non-word dictation test errors (n°)	6.08 ± 3.10 6 [4–8]	4.04 ± 2.47 3 [3–5]	**0.001**
passage dictation test errors (n°)	17.27 ± 10.40 16 [12–17]	18.08 ± 10.04 15.5 [10.25–23.75]	0.693

**Table 3:** Primary outcomes in Group A (ADHD + DSA).

	White Noise Mean ± SD and Median [IQR]	No Noise Mean ± SD and Median [IQR]	*p* Value
passage reading test speed (syl/s)	2.54 ± 1.21 2.84 [1.71–3.50]	2.33 ± 1.09 2.37 [1.78–3.16]	0.197
passage reading test errors (n°)	5.71 ± 2.52 6 [4.63–7.25]	7.92 ± 3.29 7.25 [5.88–10.50]	**0.022**
word reading test speed (syl/s)	2.35 ± 1.14 2.72 [1.65–3.28]	2.13 ± 1.04 2.30 [1.34–2.93]	0.130
word reading test errors (n°)	7 ± 6.24 4.50 [1.75–12.50]	7.67 ± 7.11 5.50 [1.50–12.50]	0.672
non-word reading test speed (syl/s)	1.57 ± 0.62 1.70 [1.27–2.05]	1.42 ± 0.58 1.59 [1.20–1.72]	0.075
non-word reading test errors (n°)	6.08 ± 4.14 6.50 [2–9.25]	10.33 ± 5.42 9 [7.50–13.75]	**0.004**
word dictation test errors (n°)	7.17 ± 6.60 5 [3–10.75]	6.58 ± 5.88 3.50 [2.75–10.75]	0.700
non-word dictation test errors (n°)	5.50 ± 3.15 6 [2.75–7.25]	3.50 ± 3 3 [2–4]	**0.009**
passage dictation test errors (n°)	17.83 ± 13.33 16.50 [11.50–17]	19.75 ± 12.47 16.5 [13–23.50]	0.482

**Table 4:** Primary outcomes in Group B (DSA).

	White Noise Mean ± SD and Median [IQR]	No Noise Mean ± SD and Median [IQR]	*p* Value
passage reading test speed (syl/s)	1.85 ± 0.75 1.69 [1.46–1.93]	1.77 ± 0.63 1.60 [1.33–2.20]	0.424
passage reading test errors (n°)	9.68 ± 3.58 10.25 [6.88–12.25]	10.18 ± 4.99 9.25 [5.88–12.38]	0.584
word reading test speed (syl/s)	1.77 ± 0.78 1.60 [1.16–2.17]	1.66 ± 0.70 1.47 [1.18–2.08]	0.251
word reading test errors (n°)	8.14 ± 6.36 7.50 [2.50–11.75]	9.57 ± 8.31 7.50 [4.25–13.75]	0.242
non-word reading test speed (syl/s)	1.16 ± 0.51 0.97 [0.83–1.18]	1.03 ± 0.34 1.01 [0.73–1.09]	**0.039**
non-word reading test errors (n°)	8.50 ± 7.48 6.50 [3.50–9.75]	9.93 ± 6.02 11 [4.50–12]	0.240
word dictation test errors (n°)	6.14 ± 6.95 3.50 [2–7.75]	6.86 ± 6.37 5.50 [3.25–6]	0.297
non-word dictation test errors (n°)	6.57 ± 3.08 5 [4–9.75]	4.50 ± 1.91 4.50 [3–6]	**0.0003**
passage dictation test errors (n°)	16.79 ± 7.56 15.5 [12.50–20.75]	16.64 ± 7.57 15 [10.25–22.50]	0.858

## Data Availability

The datasets generated and analyzed during the current study are not publicly available due to ethical and legal restrictions related to patient confidentiality, but may be available from the corresponding author on reasonable request.
